# Biomarkers in Heart Failure: A Review and a Wish

**DOI:** 10.3390/ijms26168046

**Published:** 2025-08-20

**Authors:** Laura Asta, Calogera Pisano, Adriana Sbrigata, Giuseppe Maria Raffa, Letizia Scola, Carmela Rita Balistreri

**Affiliations:** 1 Department of Neuroscience, Imaging and Clinical Sciences, Cardiac Surgery Department, University “G. d’Annunzio” Chieti-Pescara, 66100 Chieti, Italy; laura.asta@unich.it; 2 Cardiac Surgery Unit, Department of Precision Medicine in Medical Surgical and Critical Area (Me.Pre.C.C.), University of Palermo, 90134 Palermo, Italy; calogera.pisano@unipa.it (C.P.); adriana.sbrigata@gmail.com (A.S.); giuseppe.raffa@unipa.it (G.M.R.); 3 Heart Centre, IRCCS-ISMETT (Istituto Mediterraneo per i Trapianti e Terapie ad Alta Specializzazione), Via Tricomi 5, 90127 Palermo, Italy; 4 Cellular, Molecular and Clinical Pathological Laboratory, Department of Biomedicine, Neuroscience and Advanced Diagnostics (Bi.N.D.), University of Palermo, Corso Tukory, 211, 90134 Palermo, Italy; letizia.scola@unipa.it

**Keywords:** heart failure, biomarkers, multi-biomarker panels, artificial intelligence, multi-omics, targeted therapies, benefits, limitations, solutions

## Abstract

Natriuretic peptides (NPs) have significantly improved the assessment and management of patients with heart failure (HF), but they present several limitations. It is now clear that no single biomarker can adequately guide the diagnosis, prognosis, and outcomes of HF. Therefore, the use of multiple biomarkers, correlated with HF pathophysiology, may improve HF management. An exponential number of emerging biomarkers have been reported in the literature, and when combined, they demonstrate greater clinical relevance than when used alone. They are also increasingly used as targets in the development of innovative treatments, such as targeted and personalized therapies. Their identification and clinical relevance could also be facilitated by the application of artificial intelligence models and the use of multi-omics approaches. This article presents a detailed overview of emerging biomarkers, potential targets, and innovative therapies, illustrating their advantages and limitations, as well as possible solutions to overcome them, and highlighting their strong, promising potential. This could enable the prediction of the spread of this disease in the general population, enabling early diagnosis and limiting complications and mortality. The path to achieving this goal is arduous, but it is achievable. This will require further efforts by researchers and clinicians with diverse multidisciplinary expertise.

## 1. Introduction

Heart failure (HF), in the common consensus of all the worldwide HF societies, is considered, or better defined, as a cardiac syndrome, having symptoms and signs caused by structural and/or functional cardiac alterations and being characterized by the presence in circulation of high natriuretic peptide (NP) levels and/or pulmonary or systemic congestion. Consistent [1,2,3] with left ventricular (LV) ejection fraction (EF), affected cases are classified into three subtypes: (a) HF with reduced ejection fraction (HFrEF): EF ≤ 40%; (b) HF with mildly reduced ejection fraction (HFmrEF): EF between 41% and 49%; and (c) HF with preserved ejection fraction (HFpEF): EF ≥ 50% [4].

**Figure 1:**
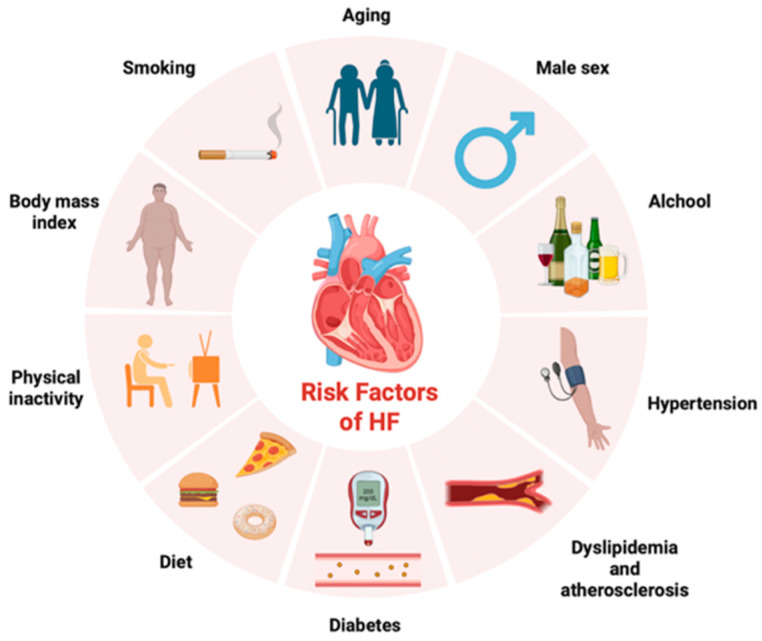
The main recognized risk factors of HF.

Today, HF represents the main health and economic challenge for countries because of its ever-increasing prevalence and incidence among our populations, particularly among the elderly. Aging constitutes one of the major risk factors of HF, followed by sex/gender with a significant rise in women [5]. Precisely, HF has raised in the last 50 years from 18.9% to 22.6% in women and from 19.1% to 25.3% in men [6]. An increased incidence of risk factors (i.e., aging, systemic arterial hypertension, smoking, obesity, diabetes, see Figure 1) or the co-presence of comorbidities (i.e., other cardiac or cardiovascular diseases) also justifies the growing concern about the enhancement of HF cases, as well as the improvement in survival after myocardial infarction onset. For example, arterial hypertension is the most common cause of HF in Latin America, Caribbean, Eastern Europe and Sub-Saharan Africa, ischemic heart disease in North America and Europe, and valvular heart disease in East Asia and Asia–Pacific countries [7].

**Figure 2:**
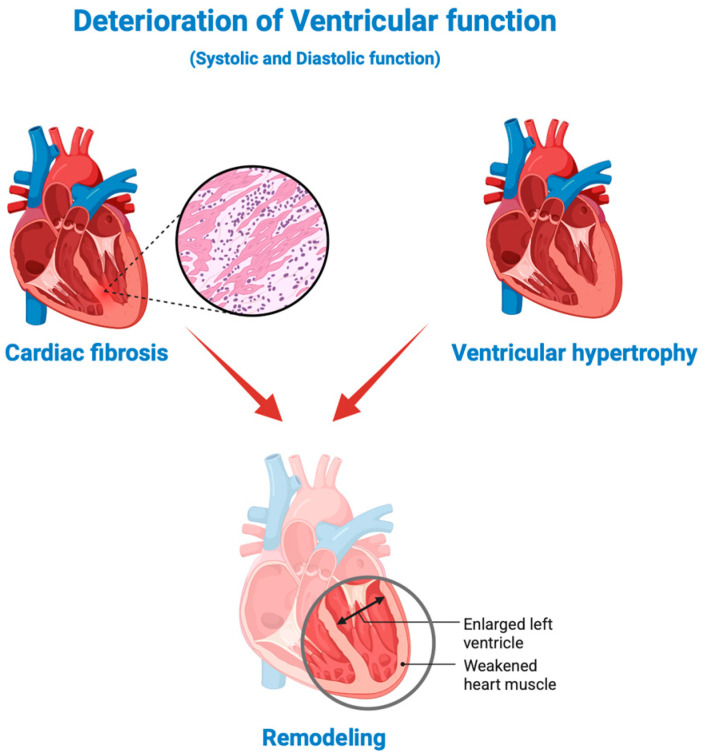
The physio-pathological alterations in HF.

The wide range of etiological causes and comorbidities related to HF onset reflect the complex pathophysiology of such disease. This makes the identification of all the cellular and molecular mechanisms involved very complex, and many remain poorly understood [8]. However, the deterioration in ventricular function constitutes the principal mechanism that causes an increase in filling pressures and a progressive reduction in cardiac output under stress, in subsequent phases, and at rest [9]. The deterioration in ventricular function results in both systolic and diastolic functional alterations. It is characterized by cardiac fibrosis, ventricular hypertrophy, and subsequent unfavorable remodeling. Such modifications can first cause diastolic dysfunction with a reduction in cardiac compliance. The diastolic dysfunction of the left ventricle, occurring due to the development of upstream pressures, affects the left atrium, pulmonary circulation, and therefore the right ventricle (Figure 2). The resulting hemodynamic alterations are responsible for the onset of the various comorbidities, including atrial fibrillation and pulmonary hypertension. These factors exacerbate the HF prognosis. In addition to diastolic alterations, systolic dysfunction occurs as consequence of the ventriculus–arterial coupling [9]. It can cause the onset of ventricular conduction delay, such as “widened QRS complex” o “broad QRS complex” (>100 ms), identified as an independent predictor of adverse outcomes [10]. Such modifications, resulting from the reduction in cardiac output, cause the activation of diverse mechanisms evocated by different organs and systems. Some of these mechanisms are compensatory, such as the activation of the sympathetic nervous system and the renin–angiotensin–aldosterone system (RAAS), as well as increased peripheral vasoconstriction [11]. In turn, they determine alterations in the NO signaling pathway, which cause variations in smooth muscle relaxation and vascular permeability, as well as the activation of the inflammatory cascade [12].

## 2. Management of HF: From Symptoms and Physical Examination to Advanced Imaging and Biomarkers

In accordance with recent guidelines [2], integrating clinical history with various clinical investigations, ranging from evaluation of signs and symptoms to physical examination, advanced imaging, and blood biomarkers, is recommended to facilitate the management of HF and to identify heart failure subtypes (phenotypes) [13]. HF symptoms (i.e., elevated jugular venous pressure, hepatojugular reflux, peripheral edema, breathlessness, orthopnea, reduced exercise tolerance and fatigue) are categorized according to the New York Heart Association (NYHA) classification. This allows the distinction as class IV of HF by considering the progressive limitation of cardiac function, determined by dyspneic symptoms. On the other hand, a longitudinal study, including 13,535 patients from the Swedish HF Registry, demonstrated that NYHA class valuation might be the more appropriate approach for clinical practice [14]. Furthermore, physical examination, such as assessment of pulmonary artery pressure by palpation, is clinically relevant when determined by right heart catheterization. This is supported by some studies. For example, Pellegrini’s group highlighted that the assessment of pulmonary arterial compliance, by quantifying the ratio between stroke volume and pulse pressure (SV/PP) in 306 patients with chronic HF caused by systolic left ventricular dysfunction and undergoing clinically guided right heart catheterization, represents a strong prognostic indicator. Imaging investigations (i.e., heart ultrasound imaging and MRI (magnetic resonance imaging) in the management of HF are well recognized. They enable HF diagnosis, the identification of causes, appropriate management, and the monitoring of therapeutic treatments [2,15]. Biomarkers remain a fundamental milestone for the diagnosis, management, and prognosis of HF. Although many biomarkers are widely used in cardiovascular disease and the management of chronic heart failure, some are specific to acute heart failure. Furthermore, several new biomarkers have recently shown promising results, particularly in acute heart failure, which can potentially improve patient management. This review will focus on the use of biomarkers in HF, highlighting the use of established biomarkers and the potential of new biomarkers to improve diagnosis, prognosis, and management.

## 3. Biomarkers in HF: From the Traditional Biomarkers, BNP and NT-proBNP, with Their Advantages and Limitations, to Emerging Biomarkers

The hemodynamic alterations that affect cardiac compliance (increased filling pressures, reduced cardiac output) and the resulting peripheral changes (increased vasoconstriction, increased vascular permeability, the activation of the RAAS, the dysregulation of the autonomic nervous system, the activation of inflammatory processes) determine the release of a series of biomarkers which, therefore, turn out to be extremely important laboratory data both for diagnostic and prognostic evaluation, as well as for the evaluation of the response to treatment. The lack of improvements in outcomes and care for patients with heart failure has been highlighted. This may be related to the inability to recognize the different pathophysiological mechanisms that occur during heart failure and the difficulty in identifying which patients have worse prognosis and require more aggressive interventions. Biomarkers can fill these gaps and their routine integration into clinical practice can improve outcomes.

### 3.1. Traditional Biomarkers: BNP and NT-proBNP with Their Advantages and Limitations

The discovery of the cardiac endocrine system, which occurred more than 30 years ago, led to the consequent detection and clinical use of peptides with natriuretic and vascular smooth muscle-relaxing activity functions. They are classified as **atrial natriuretic peptides (ANP)**, brain **natriuretic peptide (BNP)**, first isolated in porcine brain cells and only subsequently isolated in even higher doses in ventricular cardiac cells, and **C-type NP (CNP)**, mainly expressed in chondrocytes and endothelial cells exposed to cytokines [16]. In general, natriuretic peptides play a fundamental role in maintaining the volumetric balance of the cardiovascular system, counteracting pressure and volumetric overload [17]. ANP and BNP are the peptides mostly involved in this mechanism. Furthermore, ANP is, mostly stored at the intracellular level, while BNP is mostly secreted at the extracellular level. Consequently, BNP has been used as diagnostic and prognostic HF biomarker. The precursor of BNP, pro-BNP, is synthesized by cardiomyocytes after an increased cardiac preload, causing stretching. Mechanical stretching, in fact, activates signal transduction that leads to the downstream transcription and translation of a 134 amino acid precursor peptide, pre-pro-BNP [18]. Pre-pro-BNP is subsequently cleaved by two enzymes, leading to the production of the biologically active C-terminal peptide, BNP1-32, and an inactive N-terminal fragment, NT-proBNP [17].

**Figure 3:**
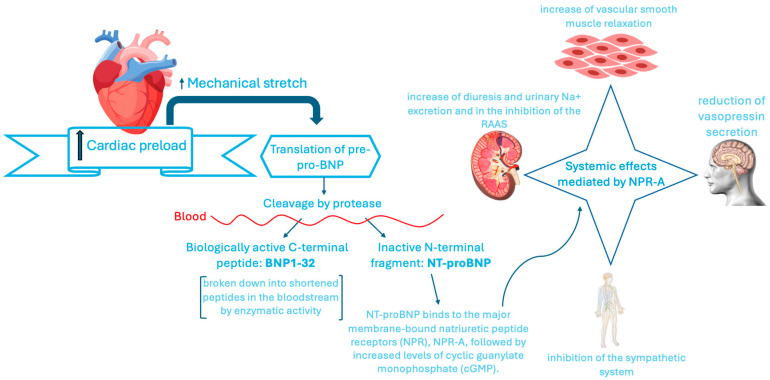
NT-pro-BNP synthesis.

The main systemic effects of BNPs are mediated by the receptor natriuretic peptide receptor A (NPRA), a receptor associated with guanylate cyclase and widely localized in the kidney, vascular smooth muscle cells, neuronal cells, and myocardium. Therefore, the receptor–ligand binding determines the activation of cGMP-dependent protein kinases (PKG), cGMP-dependent ion channels and cGMP-regulated cyclic nucleotide phosphodiesterase [19]. At the central nervous system level, the main effects consist in the increase in vascular smooth muscle relaxation (through the suppression of endothelin secretion), and in a reduction in vasopressin secretion. At the peripheral nervous system level, the effects are the inhibition of the sympathetic system, an increase in diuresis and urinary Na+ excretion, and the inhibition of the RAAS, with ther consequent effect of reduction in cardiac preload and afterload [20,21] (Figure 3).

For this reason, the secretion of the BNP hormone may be considered “emergency” secretion in the case of volume overload [22]. BNP degradation is due to two different degradation pathways: natriuretic peptide receptor C (NPR-C) and hydrolysis by neutral endopeptidase (NEP). However, the significant resistance of BNPs to NEP has been shown, unlike what happens with other natriuretic peptides, which would explain the delayed metabolism of BNPs [23]. Instead, the elimination of NT-proBNP is entirely dependent on renal activity, which is reflected in the different half-lives of the two natriuretic peptides. In fact, BNPs have a half-life of about 20 min, while NT-proBNP has one of about 1–2 h; therefore, its values display less variation than BNP but depend to a greater extent on renal function [24,25].

#### 3.1.1. NTproBNP and HF

**Figure 4:**
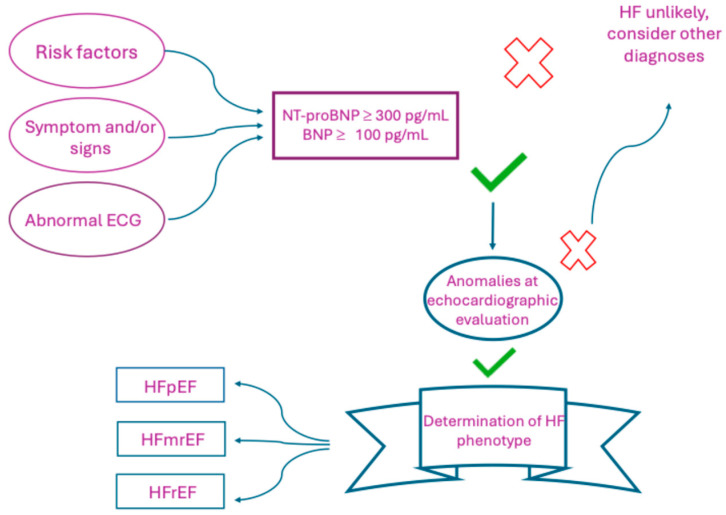
ESC Guidelines [2].

The use of BNP and NT-proBNP in HF has acquired increasing value over time, both from a diagnostic and prognostic point of view, since these natriuretic peptides are secreted exclusively by cardiac cells. However, it is essential to remember that, in addition to not being the only biomarkers used for the diagnosis of heart failure, their values, although determined by precise cutoffs for practical purposes, should be considered to a greater extent based on their trend over time [26]. According to the latest European guidelines, the cutoff values for BNP and NT-pro-BNP in support of the diagnostic hypothesis of heart failure are 100 pg/mL for BNP and 300 pg/mL for NT-proBNP or >450 pg/mL if aged <55 years, >900 pg/mL if aged 55–75 years, and >1800 pg/mL if aged >75 years [2]. In fact, current guidelines suggest a diagnostic pathway in which the dosage of BNP and NT-proBNP values only follow the results of clinical and ECG examination, even if these are obtained before the evaluation of EF (Figure 4).

Furthermore, Fang et al. highlighted the role of NT-pro-BNP as an early diagnostic biomarker in patients with diabetes in the absence of a clear clinical expression of heart failure (subclinical cardiovascular disease). In fact, in a population of 10,304 subjects not affected by evident cardiovascular disease with or without diabetes, they demonstrated how in diabetic subjects’ high values of NT-proBNP were independently associated with all-cause mortality [27]. Similarly, in a previous meta-analysis, the Natriuretic Peptides Studies Collaboration demonstrated that in the absence of a defined cardiovascular pathology, the dosage of NT-proBNP values could predict the onset of heart failure, coronary artery disease, and stroke [28]. The diagnostic value of BNPs and NT-pro-BNP has not only been recognized at the European level. However, a broader consensus, including American and Japanese task forces, considers the two markers essential for reaching the diagnosis of heart failure, even though the definition of the disease itself does not include natriuretic peptides. It is, however, necessary to underline that the increase in BNPs and NT-proBNP may also depend on other comorbidities such as chronic renal failure or atrial fibrillation, the latter frequently being associated with heart failure [29]. Furthermore, it has been highlighted that the value of NT-proBNP should also be correlated with sex and BMI [30]. Indeed, a U-shaped relationship between BMI and NP levels has been demonstrated, with higher NP levels at both extremes of the BMI distribution [31]. Severe obesity is associated with a significant increase in NT-proBNP levels, determined by the increased expansion of plasma volume, with greater biventricular remodeling and a consequent increase in filling pressures. This identifies a subcategory of patients with HF, a preserved ejection fraction, and a higher risk of cardiovascular events [31,32,33]. In a population of 18,356 individuals without previous cardiovascular disease, Welsh et al. demonstrated that female patients were 7 times more likely to have levels higher than the cutoff value of NT-proBNPs (≥125 pg/mL) after adjusting for age [34]. This evidence would suggest the need for an increase in the cutoff to avoid diagnostic errors, around 400 pg/mL. However, this would lead to a diagnostic delay in patients with an already evident diagnostic suspicion of heart failure [34]. Finally, the increase in NT-proBNP values with advancing age can be easily explained by the progressive deterioration of ventricular function, the increased incidence of cardiovascular diseases, and the reduction in renal function [30]. This leads to the affirmation that NT-proBNP values cannot be considered as absolute values but must be related to the time of the onset of the clinical picture of HF. For this reason, different cutoffs are used in cases where heart failure has an acute onset or in cases of outpatient management [35]. In particular, in cases of acute-onset heart failure, ICON-RELOADED (International Collaborative of NT-proBNP: Re-evaluation of Acute Diagnostic Cut-Offs in the Emergency Department), revised in 2018, developed age-related cutoff values in order to confirm or exclude the diagnosis of acute-onset heart failure (the odds of which are significantly higher than those of chronic patients, as the heart in the acute phase is subjected to greater traction of the fibers and therefore undergoes a massive synthesis of NT-proBNP): this research was performed in patients aged <50 years for values ≥450 pg/mL, in patients aged between 50 and 75 years for values ≥900 pg/mL, and in patients aged >75 years for values ≥1800 pg/mL. However, in case of a significant diagnostic suspicion of HF, clinical investigations and instrumental diagnosis are required. Values ≥5000 pg/mL are indicative of a high diagnostic suspicion of HF and are also correlated with a worse prognosis [35,36]. In the absence of acute exacerbation, the cutoff value to raise a diagnostic suspicion of HF is a dosage of NT-proBNP ≥125 pg/mL, also established by the European guidelines. However, no distinction was made based on age. However, the practical algorithms for the early diagnosis of HF propose defining a high risk of HF for values ≥125 pg/mL in patients aged under 50 years, for values ≥250 pg/mL in patients aged between 50 and 74 years, and for values ≥500 pg/mL in patients aged ≥75 years. Values ≥2000 pg/mL are indicative of a high risk of heart failure, requiring further diagnostic investigations (echocardiogram, MRI) within two weeks [35]. From the identification of these cutoff values, it emerges that there is a significant “gray area” in which the diagnosis is rather indeterminate (for example in patients aged over 50 years and with values between 450 and 900 pg/mL) and which requires further diagnostic tests, in addition to the adjustments for sex, BMI, and renal function, as discussed above. Ianos et al., similarly, highlighted how high NT-proBNP values (above 2379 pg/mL), after adjusting for demographic and clinical covariates, were significantly associated with increased odds of advanced HF and how the measurement of NT-proBNP levels had the highest sensitivity and specificity values (area under the receiver operating curve (AUC) 0.73, 95% C.I. 0.63–0.82), demonstrating not only the diagnostic but also the prognostic role of NT-pro-BNP [37]. Similarly, Nguyen et al. in their prospective study correlated NT-pro-BNP values with the rate of 90-day post-discharge events in patients with HF displaying a reduced ejection fraction, demonstrating that NT-proBNP had the highest predictive value, both in terms of univariate Cox proportional hazard analysis [HR (95%CI) = 2.49 (1.41–4.40), *p* value 0.002] and multivariate analysis [HR (95%CI) = 2.36 (1.31–4.24) *p* value 0.004] [38]. Finally, Ammar et al. in their systematic review and meta-analysis of the reading also addressed the correlation between NT-proBNP values and the risk of adverse events in patients with HF with a preserved ejection fraction. In particular, the authors, integrating the results of 22 articles published between 2008 and 2024 that included a population of 10,158 patients with HFpEF aged between 44 and 82 years, demonstrated a close correlation between high NT-proBN values and the risk of mortality [HR of 1.65 (95% CI: 1.55–1.76)] [39]. This further supports the solidity of using NT-proBNPs for diagnostic purposes in HF and for establishing its prognosis. Table 1 summarizes the main advantages and limitations of the clinical use of NT-proBNPs.

#### 3.1.2. MR-pro-ANP: Another Biomarker Prevalently Associated with the HF Diagnosis

Like B-type peptides, a rapid increase in the blood levels of atrial natriuretic peptide (ANP) also occurs after cardiac stretch, but, due to its half-life of only 2–5 min, linked to the action of neprilysin, quantifying this is problematic [40]. By contrast, its immediate precursor protein, pro-ANP, which is more stable and has a longer half-life, can be quantified in serum samples. Consequently, a mid-regional pro-peptide assay for ANPs (MR-pro-ANP) enabled such quantification and its application in HF [41]. MR-pro-ANP was first used as a diagnostic biomarker in the BACH (Biomarkers in the Acute Heart Failure) study [41]. However, its comparison with BNPs or NT-pro-BNP shows in that study, as well as in the PRIDE study [42], that it does not perform significantly better: with a MR-pro-ANP cutoff point of ≥120 pmol/L and a sensitivity of 97%, a specificity of 60% with an accuracy of 74% compared to the BNP cutoff point of 100 pg/mL, and a sensitivity of 96%, a specificity of 62%, and an accuracy of 73%. In chronic heart failure, the GISSI-HF study [43] evaluated the ability of MR-pro-ANP to predict stable chronic heart failure. The results obtained showed that MR-pro-ANP values ≥278 pmol/L had better prognostic accuracy for 4-year mortality compared to other new and established biomarkers included in the study (AUC = 0.74; 95% CI, 0.70–0.76). Better results were obtained by adding BNPs or NT-pro-BNP. This analysis was shown to improve the diagnostic performance of MR-pro-ANP for chronic heart failure with an improvement in AUC and net reclassification index (NRI), which are statistical methods used to assess the ability of a biomarker to adequately classify a patient’s risk [44]. However, it has been reported that the performance of MR-pro-ANP is still reduced in patients with BNPs and NT-pro-BNP values within the ‘gray zone’ [45]. Furthermore, another limitation is represented by the MR-pro-ANP test, which shows the same interferences as the BNPs and NT-pro-BNP tests. Therefore, limited data on the diagnostic potential of MR-pro-ANP have been provided so far, and the use of MR-pro-ANP is still largely left to research. Our hypothesis is that blood levels of MR-pro-ANP largely reproduce pathophysiological processes, such as those of BNPs and NT-pro-BNP. Therefore, it is not surprising that MR-pro-ANP is inappropriate in terms of efforts to significantly facilitate the diagnosis of HF [46]. A perfect biomarker able to integrate such molecules would reflect other pathophysiological processes occurring in HF [45].

### 3.2. Troponins as Myocardial Damage Biomarker

**Cardiac troponins (cTn)** [47] are regulatory proteins of cardiomyocytes, which contribute to the calcium-mediated interaction between actin and myosin. They constitute a protein complex, consisting of three subunits: troponin T, troponin I, and troponin C. Cardiac troponins represent the typical biomarkers of the management of acute coronary syndrome. However, they are also recognized as useful prognostic biomarkers of HF. Accordingly, increased blood levels of cTn in HF are ascribed to myocardial stress and cardiomyocyte death related to the persistent subendocardial ischemia [48]. Furthermore, cardiac troponins, as biomarkers of essential myocyte dysfunction, could also predict new HF, since low-level chronic myocyte damage may appear in individuals before the clinical onset of HF. Elevated circulating cTn levels have been reported to be associated with advanced HF, poor prognosis, and mortality risk. Elevated cardiac troponin (cTn) levels are also associated with an increased risk of developing heart failure (HF) in individuals who have experienced prior acute myocardial infarction, as higher cTn concentrations tend to correlate with a greater likelihood of HF onset [48]. In patients with acute HF, those with elevated baseline cTn levels are more frequently hospitalized, have increased intensive care unit admissions, and have higher in-hospital mortality. In individuals with chronic HFrEF, elevated cardiac troponin (cTn) levels have been associated with greater long-term mortality and an increased likelihood of rehospitalization due to HF [49,50]. However, the increase in plasma cTn values varies by gender and race, being greater in males and blacks. In addition, the prevalence of cardiovascular disease, advanced age, hypertension, left ventricular hypertrophy, and chronic kidney disease may lead to an increase in cTn values or other clinical conditions not related to myocardial damage such as sepsis, acute respiratory distress syndrome, and stroke [51].

## 4. Emerging Biomarkers: From Diagnostic to Prognostic and Therapeutic Purpose

Recent evidence highlights that systemic inflammation and oxidative stress-related pathways play a crucial role in the pathophysiology of HF compared to the activation of the sympathetic nervous system and RAAS [52,53,54,55,56]. This led to the study of several pathophysiological pathways and the subsequent identification of several potentially useful biomarkers. They could support clinical decision-making in HF screening and diagnosis, prognostic stratification, and therapeutic guidance. On the other hand, B-type neurotransmitters (i.e., BNPs and NT-pro-BNP, as extensively described above) represent the gold standard in HF management, but in recent years other molecules have been shown to have value in HF assessment [47,54,55,57,58,59]. However, we cotemporally underline that the number of biomarkers evaluated in heart failure has grown exponentially, but only a few of them have had their efficiency demonstrated with sufficient evidence to justify their use in clinical practice [47,53,54,55,57,58]. Furthermore, most studies have tested new biomarkers exclusively in the prognostic stratification of heart failure patients, while in the literature there are few data on their use as screening, diagnostic, and treatment guidance tools. This last aspect appears particularly relevant given the lack of reliable analytical indices that can be used as a reference for the titration of heart failure therapy. This could lead to the combination of multiple biomarkers belonging to different pathophysiological pathways and facilitate both the optimization and the personalization of clinical HF management. The integration of different omics technologies into such investigations, although still far from a possible routine clinical application, could contribute in the future to defining the clinical phenotype of individual heart failure patients much more precisely. Below, we report a large list of emerging biomarkers, dividing them according to their molecular characteristics and functions [47,53,54,55,57,58].

### 4.1. Biomarkers of Neuro-Hormonal Activation

HF is a complex and heterogeneous condition driven by various interrelated pathophysiological processes. Key features such as fluid accumulation, vasoconstriction, and adverse cardiac remodeling arise primarily from neurohormonal imbalances, including the activation of both the renin–angiotensin–aldosterone system (RAAS) and the sympathetic nervous system. This article explores several molecules, associated with these pathways, that may serve as potential biomarkers for heart failure. Notably, plasma norepinephrine was one of the earliest indicators identified as a reliable predictor of heart failure outcomes, as first demonstrated by Cohn and colleagues [60]. This was proven in some pharmacological studies [61,62]. More recent research, however, has indicated that norepinephrine does not offer superior prognostic value when compared to BNPs [62]. In addition to catecholamines, chromogranin A and B, contained in the granules of neuroendocrine cells, have also been shown to exhibit increased expression proportionally to HF severity. A limited-scale study suggested that chromogranin A could serve as a useful marker for predicting mortality in individuals with chronic heart failure [63]. Within the RAA system, several components have been studied as potential prognostic biomarkers in heart failure, with plasma renin activity (PRA) showing the most significant findings [64]. In a study of 996 patients with chronic HF, PRA was found to be an independent predictor of cardiac death, providing additive value to NT-proBNP and LVEF [65]. Another molecule designated for inclusion in this group is adrenomedullin (ADM), a hormone primarily produced by the adrenal medulla, as well as the heart, lungs, and kidneys, in response to volume or pressure overload [66]. It exhibits potent vasodilatory actions, positive natriuretic and inotropic effects, as well as cardioprotective action. Although plasma levels of adrenomedullin (ADM) rise in HF, its clinical measurement is limited by a short half-life and strong affinity for transport proteins [41]. Mid-regional pro-adrenomedullin (MR-proADM), a stable fragment derived from the precursor of adrenomedullin, is easier to measure and has been investigated as a prognostic biomarker in heart failure. In the Biomarkers in Acute Heart Failure (BACH) study, MR-proADM outperformed BNPs in predicting 90-day survival in patients with acute heart failure, demonstrating greater accuracy (74% vs. 62%, *p* < 0.001). These findings were further supported by a sub-analysis of the PRIDE (N-terminal Pro-BNP Investigation of Dyspnea in the Emergency Department) study, which identified MR-proADM as the most reliable predictor of mortality within the first year following an acute HF diagnosis. In contrast, MR-proANP and NT-proBNP provided superior prognostic value beyond the one-year mark [41]. In the Australia-New Zealand Heart Failure Study including 297 patients with chronic HF, MR-proADM levels above the median predicted an increased risk of mortality (relative risk [RR] 3.92, 95% confidence interval [CI] 1.76–8.7) and HF hospitalization (RR 2.4, 95% CI 1.3–4.5) at 1.5 years, independent of clinical and echocardiographic parameters. MR-proADM was also found to be a good predictor of 1-year survival in another study of 501 patients with chronic heart failure with an area under the curve (AUC) like that of NT-proBNP (*p* = 0.3) [42,67,68].

Vasopressin has also been studied as a potential biomarker of HF. It is a hormone released from the hypothalamus in response to hyperosmolarity or hypovolemia with antidiuretic and vasoconstrictor activity, [69]. In HF, inappropriate vasopressin release is largely triggered by baroreceptor overstimulation resulting from decreased cardiac output, which the body interprets as a hypovolemic condition. Copeptin, the C-terminal portion of the vasopressin precursor, is more stable and easier to measure, making it a valuable biomarker of HF. Patients with elevated systemic copeptin levels in the BACH study had increased 3-month mortality (hazard ratio [HR] 3.85, 95% CI 1.83–8.09; *p* < 0.001) and pulmonary and peripheral congestion, especially if they had hyponatremia [69]. Copeptin was reported to be a good predictor of all-cause mortality (RR 2.64, 95% CI 2.09–3.32) with a performance comparable to that of NT-proBNP in a meta-analysis of 4473 patients with acute and chronic heart failure [70].

Molecules derived from the endothelium, such as endothelin-1 (ET-1), also belong to this category. ET-1 is synthesized by vascular endothelial cells in response to shear stress (i.e., the effect of flow friction along the longitudinal axis of the vessel on the intima), neurohormonal stimulation, and inflammation, exerts vasoconstrictive, pro-inflammatory, and pro-oxidative biological effects, and favors cardiac remodeling. ET-1, precursor synthesized by endothelium, is then converted into ET-1 by the circulating endothelium [71].

Finally, this group includes urocortin-1. It is part of the family of corticotropin-releasing factors and produced in numerous peripheral tissues, such as the heart, but it primarily produced in the central nervous system. Urocortin-1 has vasodilatory, cardiostimulatory, and cardioprotective properties. While circulating urocortin-1 levels are elevated in HF, they do not provide added diagnostic or prognostic benefit beyond that of NT-proBNP [72].

### 4.2. Biomarkers of Fibrosis and Cardiac Remodeling

Fibrosis and cardiac remodeling result from chronic stress on the heart, resulting in impaired myocardial function and an increased risk of arrhythmias. Here, we report some of the molecules involved in these mechanisms, which might be used as HF biomarkers [47,73,74,75].

**Galectin-3 (Gal-3)**, a member of the Galectin protein family, was the first identified in this category and is known for its role in promoting inflammation and fibrosis. Acute or chronic cardiac injury cause inflammation, and the innate immune cells involved release cytokines that activate fibroblasts and myofibroblasts, depositing collagen and evocating cardiac fibrosis. Cardiac injury and fibrosis represent the two remarkable mechanisms in the HF onset [76,77,78,79]. Gal-3 levels enhance progressively with age and tend to be slightly higher in women than in men. In addition, Gal-3 also raises in conditions of systemic inflammation and renal failure. Thus, it is not HF-specific, even if its expression in HF is related to pro-inflammatory cytokines. While plasma levels of Galectin-3 are not effective in diagnosing heart failure, they have shown value in predicting short-term mortality and the likelihood of rehospitalization. Additionally, Gal-3 has been proposed as a potential marker for forecasting all-cause mortality and heart failure-related hospital admissions (RR 3.4, 95% CI 2.09–3.32) [76,77,78,79]. The prognostic power of Gal-3 is more significant in HFpEF than in HFrEF [76,77,78,79].

Another potential member of this group is **suppression of Tumorigenicity-2 (ST2)**, which belongs to the interleukin-1 receptor family. ST2 exists in two main forms: a transmembrane receptor (ST2L) and a soluble variant, known as sST2. The soluble form acts as a decoy receptor and can be measured in the bloodstream. Its biological ligand is interleukin-33 (IL-33) [80,81,82,83,84,85]. The interaction between ST2L and IL-33 is implicated in communication between cardiomyocytes and fibroblasts within the myocardium. This pathway becomes upregulated in response to myocardial injury, where it appears to exert a protective effect on the heart. The soluble isoform, sST2, can bind IL-33 with a higher affinity, consequently competing with the interaction between IL-33 and ST2L. This results in an effect opposite to the cardioprotective one [80,81,82,83,84,85]. sST2 is produced predominantly in extra-cardiac regions in response to the most common elements of HF, such as hemodynamic overload, inflammation, and profibrotic stimuli. Since sST2 is not a cardiac-specific marker, it cannot be used for the diagnosis of acute heart failure, even if its levels are useful in prognostic stratification [80,81,82,83,84,85]. A meta-analysis involving 4835 patients with acute heart failure found that sST2 levels measured at both admission and discharge were significant predictors of all-cause mortality (hazard ratios of 2.46 [95% CI: 1.80–3.37] and 2.06 [95% CI: 1.37–3.11], respectively) as well as cardiovascular mortality (hazard ratios of 2.29 [95% CI: 1.41–3.73] and 2.20 [95% CI: 1.48–3.25], respectively) [86]. In contrast, sST2 concentration at discharge predicted rehospitalization for heart failure (HR 1.54, 95% CI 1.03–2.32). The repeated quantification of sST2 is particularly important. In a study involving 150 patients with acute heart failure, changes in sST2 levels during hospitalization were found to predict 3-month mortality independently of BNP or NT-proBNP concentrations. Similarly, the TRIUMPH study—a cohort of 496 patients with acute heart failure monitored through seven serial blood samples over a one-year follow-up—demonstrated that baseline sST2 levels were associated with the risk of all-cause mortality or heart failure-related hospitalization (HR per 1 SD increase in log_2_[sST2]: 1.30, 95% CI: 1.08–1.56). Notably, repeated measurements of sST2 offered even stronger prognostic value (HR per 1 SD increase in log_2_[sST2]: 1.85, 95% CI: 1.02–3.33), independent of serial NT-proBNP values [86,87]. The significance of sST2 in the prognostic assessment of chronic heart failure is even greater, as demonstrated by multiple studies and supported by a recent meta-analysis. Its prognostic value in chronic HF is independent of biomarkers such as NT-proBNP and high-sensitivity troponin T (hs-TnT), and it is less affected by patient age compared to these markers [86,87]. The prognostic accuracy of sST2 is comparable in both heart failure with reduced ejection fraction (HFrEF) and preserved ejection fraction (HFpEF), and it outperforms Galectin-3 (Gal-3) in patients with chronic heart failure. Additionally, sST2 has been identified as an independent predictor of reverse cardiac remodeling. Reflecting its clinical utility, sST2 has been incorporated into the ST2-R2 score, which includes the following criteria: sST2 levels below 48 ng/mL (3 points), non-ischemic heart failure etiology (5 points), the absence of left bundle branch block (4 points), a heart failure history of less than one year (2 points), left ventricular ejection fraction (LVEF) under 24% (1 point), and beta-blocker therapy (2 points). The American College of Cardiology/American Heart Association (ACC/AHA) guidelines recommend measuring sST2 as part of the prognostic evaluation in chronic HF [86,87]. On the contrary, ESC guidelines advise against its use in clinical practice. It is important to note that the majority of key studies investigating the role of sST2 in both acute and chronic heart failure were conducted and published after the release of the current clinical guidelines. As a result, there is still no universally accepted prognostic cutoff value for sST2 in chronic HF. Nevertheless, a recent meta-analysis proposes 28 ng/mL as a potential threshold, while other studies have suggested a higher cutoff of 35 ng/mL [86,87].

**Growth/differentiation factor 15 (GDF-15)**, also known as macrophage inhibitory cytokine-1 (MIC-1), may also be considered part of this group, as it has shown a significant association with HF [88,89]. Cellular stressors related to inflammation, myocardial ischemia, and cancer drive GDF-15 expression. In HF, elevated levels of GDF-15 have been independently linked to reduced exercise capacity and a poorer quality of life. These levels rise progressively across all stages of the disease, including asymptomatic stage B. Moreover, in patients with HFpEF, the combined assessment of GDF-15 and NT-proBNP enhances the ability to distinguish affected individuals from healthy controls [88,89]. In fact, the elevated levels of GDF-15 can be detected as early as 90 days before hospital admission; however, due to its pleotropic nature, the exact physiological role has not been fully elucidated. It is also known that GDF-15 is positively correlated with left ventricular remodeling [88,89], although numerous studies have shown that GDF-15 exhibits an antihypertrophic effect through the SMAD signaling pathway. Its association with adverse events in heart failure calls into question its role in controlling or contributing to the pathogenesis of heart failure. Numerous lines of evidence support the protective and antihypertrophic effects of GDF-15 on the heart. Furthermore, GDF-15 appears to have an anti-inflammatory effect, but conflicting data have also emerged regarding its cardioprotective role. Therefore, further studies are essential to fill these gaps and apply this information to the clinical management of HF [88,89].

In HF, an alteration in the expression of several extracellular matrix degradation products, such as **MMPs and TIMPs**, is also observed. Results, although variable in individual studies, have evaluated the use **of MMP and TIMP** as diagnostic or prognostic biomarkers in HF [90,91]. Research conducted by Zile et al. showed that elevated levels of MMP-2, TIMP-4, and procollagen type III amino-terminal peptide (PIIINP), along with reduced MMP-8 concentrations, were predictive of HFpEF, with an area under the curve (AUC) of 0.79 [90]. A subanalysis of the RALES (Randomized Evaluation of Aldactone Study) trial found that baseline PIIINP levels above 3.85 μg/L were associated with worse clinical outcomes. Notably, a reduction in PIIINP levels was only observed in patients treated with spironolactone, supporting its antifibrotic properties. Additionally, the prognostic benefits of spironolactone were only significant in individuals with elevated baseline levels of collagen degradation markers. Similarly, a subanalysis of the PARAGON-HF (Prospective Comparison of ARNI With ARB Global Outcomes in HF With Preserved Ejection Fraction) trial demonstrated that treatment with sacubitril/valsartan led to a notable decrease in certain extracellular matrix degradation biomarkers, including TIMP-1 and PIIINP, when compared to valsartan alone after 16 months of follow-up [92].

Another potential biomarker of fibrosis of more recent discovery is Fibroblast Growth Factor 21 (FGF21) [93,94,95]. This polypeptide ligand is mainly synthesized by cells in the liver and adipose tissue. Its traditional function is as a hormonal regulator of metabolism through endocrine, paracrine, and autocrine mechanisms. Its expression is also stimulated by various cardiac stresses. Elevated serum levels of FGF21 are closely associated with left ventricular systolic dysfunction, and individuals with higher concentrations face a greater risk of cardiac mortality compared to those with lower levels. In addition, its levels are not related to other comorbidities. This has led to assuming the existence of a link between the liver and the heart, with the liver being its principal producer. In this context, increased FGF21 levels may reflect a protective compensatory response, triggered by the congestive liver disease commonly seen in advanced stages of HF. However, further research is needed [93,94,95].

More recent discoveries include **osteopontin (OPN)**, **syndecan-4 (SDC-4)** and **myostatin (MSTN) [49,96]**. OPN is a signal transduction protein associated with the extracellular matrix, and its expression is regulated by biomechanical stress. As a result, OPN production becomes particularly prominent in the heart following myocardial infarction and in cases of heart failure due to dilated cardiomyopathy. This justifies the quantification of plasma OPN levels in HF. This process demonstrates that it is an excellent prognostic biomarker [97]. SDC-4, a transmembrane glycoprotein belonging to the syndecan family, plays roles in signal transduction, tissue repair, angiogenesis, and focal adhesion. In heart failure, elevated SDC-4 levels have been significantly linked to left ventricular hypertrophy. Myostatin (MSTN), another extracellular matrix (ECM) signaling molecule, functions as a negative regulator of muscle growth and mass [98]. Serum levels of myostatin (MSTN) have been found to rise in parallel with heart failure severity and clinical deterioration. In patients with chronic heart failure, elevated MSTN concentrations are significantly linked to reduced survival and an increased rate of rehospitalization [99,100].

Lastly, several proteins involved in collagen turnover and extracellular matrix (ECM) regulation—such as **endotrophin, thrombospondin-2 (THBS-2), ADAMTS-like protein 2 (ADAMTSL2), the large ECM protein SVEP1** (sushi, von Willebrand factor type A, EGF, and pentraxin domain-containing 1), and **vascular endothelial growth factor-C (VEGF-C)**—have all been linked to cardiac remodeling and the pathophysiology of HF [49,96,101]. There might be other biomarkers.

### 4.3. Biomarkers of Inflammation and Oxidative Stress

HF is also characterized by a chronic subclinical inflammatory state that is self-sustaining from progressive cellular damage and is partly responsible for progressive cardiac remodeling. Inflammation in HF can be caused by direct damage to cardiomyocytes (e.g., ischemia, pressure overload) or rather the presence of comorbidities that determine a systemic inflammatory state, a mechanism most likely crucial for the development and progression of HFpEF. The first demonstration of an elevation of **C-reactive protein (CRP)** in HF dates is linked to a work conducted in 1953. Since then, multiple studies have emphasized the prognostic relevance of inflammatory markers such as C-reactive protein (CRP), tumor necrosis factor-alpha (TNF-α), and interleukin-6 (IL-6) in heart failure. These cytokines play a key role in the inflammatory pathways underlying HF and have also been associated with an increased risk of developing the condition, particularly in the elderly. However, their clinical utility remains limited due to their low specificity for cardiovascular diseases, including HF, and the absence of effective anti-inflammatory treatments specifically approved for HF [102,103,104].

Recently, the attention has been focused on **carbohydrate antigen-125 (CA125)**. Accordingly, the sufficient literature data currently reports that CA125 has a potential role in the clinical management of patients with acute HF, as a prognostic, congestion biomarker, and in the monitoring of decongestion therapy. However, CA-125 is universally known for its use in ovarian cancer, as well as in other processes. It is involved in fluid and cell transport, inflammation, tissue repair, and the well-known tumor dissemination. For example, N-glycans associated with CA-125 have been shown to modulate immune responses. In line with this role, CA-125 has been noted to suppress the activity of natural killer cells by interacting with several proteins. In the HF case, systemic levels of CA-125 are augmented, even if the exact mechanisms involved are not entirely identified. The involvement of Ca-125 in HF was evidenced for the first time by Nagele and coworkers. In two studies, they reported increased circulating levels of numerous tumor biomarkers, including CA 125, in HF patients before and after heart transplantation [105,106]. Since then, other researchers have examined the role of this molecule in heart failure. Romina’s group has recently reviewed all studies of CA-125 in HF in a systematic review that included data from 170 studies. In many of these, they authors highlighted the close association of CA-125 with (a) the congestion observed in acute HF, (b) the high rates of mortality and (c) readmission at 6 months of follow-up after discharge from acute HF, and (d) also the role of CA-125 in guiding HF therapy [107]. They also reported that some clinical studies have examined several peculiarities of CA-125, which make it even better than NT-proBNP in different scenarios of acute HF. However, the mechanisms underlying increased serum CA-125 in patients with congestive heart failure are not yet fully understood. Therefore, CA-125 is a promising biomarker of congestion in the setting of acute heart failure, with a role in risk stratification, monitoring, and therapeutic guidance. However, further studies are needed to implement CA-125 in clinical practice, especially to establish a reference range and define appropriate algorithms for diagnosis and treatment monitoring. Serum CA-125 levels, although commonly available, should be used in combination with clinical manifestations, other biomarkers (such as NT-proBNP), ultrasound, and other multimodal methods, for improved clinical efficacy in screening and early diagnosis [105,106].

Recently, the **CRP/albumin ratio (CAR)** has also been described as an important prognostic biomarker in chronic HF. In patients with chronic heart failure, CAR has been associated with an unfavorable clinical picture, characterized by increased left ventricular end-systolic volume (LVESV), elevated pulmonary artery systolic pressure (PAPS), and reduced tricuspid annular plane excursion (TAPSE). The inflammatory etiology of chronic heart failure may explain this association; in chronic heart failure, inflammatory mediators and pro-inflammatory cytokines induce diastolic and systolic dysfunction of cardiac muscle cells and thus their atrophy and remodeling, which are pathognomonic features of this form of HF [108,109,110,111,112].

Recent studies have also shown that the **neutrophil-to-lymphocyte ratio (NLR)** could be related to adverse outcomes in patients with HF [113,114,115,116]. Accordingly, a recent meta-analysis, including a total of 15 studies with 15,995 patients affected by acute HF, evaluated whether NLR could predict mortality. By stratifying the patients based on a cutoff NLR, it has been observed that high NLR quartiles were associated with a significantly higher in-hospital mortality [HR 1.54, 95% CI (1.18–2.00), *p* < 0.001] and long-term all-cause mortality [HR 1.61, 95% CI (1.40–1.86), *p* < 0.001] compared to the group with reduced NLR quartiles. Furthermore, comparing the highest and lowest NLR quartiles, it emerged that cases with the highest NLR quartiles had a significantly higher risk of long-term all-cause mortality [HR 1.77, 95% CI (1.38–2.26), *p* < 0.001]. Thus, it can be concluded that elevated NLR levels have predictive value in the risk of short- and long-term mortality and therefore, NLR can be used as a biomarker of adverse outcomes in patients with acute HF [115].

Another typical mechanism of HF is increased oxidative stress, related to mitochondrial dysfunction. Reactive oxygen species (ROS) damage cellular structures and promote the initiation and perpetuation of inflammatory processes, generating a vicious circle. Dosing is made difficult by the intrinsic instability of ROS. Molecules that interact with reactive oxygen species (ROS), particularly those with antioxidant properties, serve as useful indicators of oxidative stress. Among them, **myeloperoxidase (MPO)** has been investigated as a potential biomarker in heart failure, showing promising results for prognostic stratification. However, its clinical application remains limited due to the relatively small number of studies conducted to date. MPO is an enzyme secreted by leukocytes as part of the inflammatory response. In patients with chronic heart failure, elevated plasma MPO levels have been observed and shown to correlate significantly with both BNP concentrations and NYHA functional classes in one study. Another investigation involving 667 individuals presenting with acute dyspnea found that MPO levels were elevated in both cardiac and non-cardiac cases of dyspnea and independently predicted one-year mortality in patients with cardiac dyspnea, performing comparably to BNPs in this context [117,118,119].

### 4.4. Clinical Viewpoint: Considerations and Limitations

Fibrosis and cardiac remodeling represent two fundamental mechanisms in the pathophysiology of HF [73,74,120,121]. Currently, however, most of the described biomarkers have limited clinical applicability, although at the same time they have quite significant uses in efforts to identify pathways associated with HF onset and progression. Some studies using MMP inhibitors have not shown unequivocally positive results. However, the above-described biomarkers, as well as those of inflammation and oxidative stress, all appear to represent very promising therapeutic targets in both acute and chronic HF settings. Additionally, the increasing attention paid to the role of the lymphatic system in acute heart failure highlights the potential clinical value of biomarkers related to lymphatic dysfunction, such as vascular endothelial growth factor-C (VEGF-C) [122,123]. There is growing clinical rationale to support the use of biomarkers of lymphatic dysfunction—such as members of the VEGF family—as indicators of peripheral congestion. Unlike natriuretic peptides, which primarily reflect intracardiac pressure, these biomarkers may better represent interstitial fluid accumulation. Therefore, additional research is needed to validate their role and integrate them into routine clinical practice [124].

**Figure 5:**
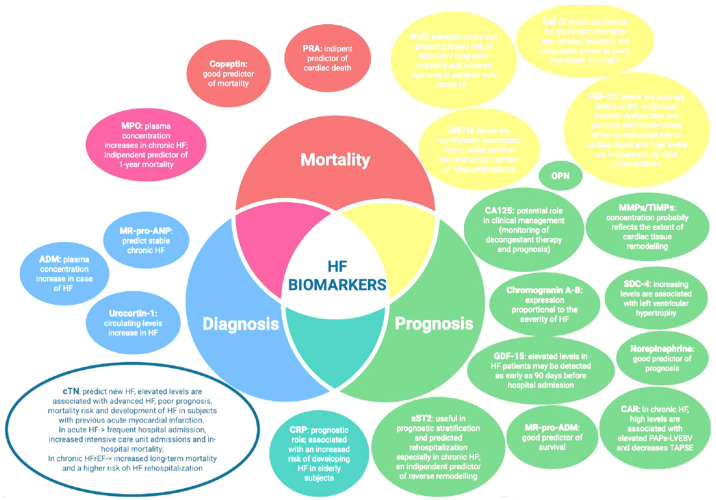
The main advantages and limitations of clinical use of HF biomarkers.

The biomarkers discussed so far with their relative advantages and limitations are summarized in Figure 5.

Table 2, instead, indicates the suggested association between HF phenotype and typical and atypical diagnostic biomarkers.

## 5. Other Promising Biomarkers: Biomarkers of Negative HF Outcomes

Patients with HF have several comorbidities, from iron deficiency [125,126,127] to endocrine–metabolic alterations [128] and bone marrow, renal, and hepatic function alterations [129,130,131,132]. Therefore, the use of appropriate biomarkers could facilitate the evaluation of HF outcomes. Emerging research is proposing the use of many biomarkers, which we describe below, by highlighting their benefits and limitations.

### 5.1. Iron Deficiency

Iron deficiency (ID) is one of the most common HF comorbidities [133]. Accordingly, 30–60% of patients with HF are affected by ID, particularly the cases with chronic and anemic form [134]. ID is more prevalent in women with HF and a more advanced stage of severity. In fact, ID significantly correlates with higher levels of natriuretic peptides and NYHA classes III or IV [134]. Recent studies have also reported that ID is prevalent in 50–70% of cases with acute forms of illness. ID is a serious comorbidity because it highlights both the anemic condition and the presence of other alterations in patients, including mitochondrial dysfunction and impaired energy processing, as alterations of reactive oxygen species and abnormalities of the immune response [134]. This also explains the attention paid to ID in HF as an effective target for therapeutic interventions. Iron deficiency is generally classified into two types: absolute and functional. Absolute iron deficiency reflects depleted or absent iron stores in the body, whereas functional iron deficiency occurs when iron stores are adequate but their availability for essential physiological processes is insufficient [135]. The downregulation of ferroportin 1, a protein involved in the efflux of iron from enterocytes, macrophages, and hepatocytes into the extracellular space and bloodstream, may be the cause of poor iron availability in functional ID [125,126,127].

The underlying reasons for the high prevalence of iron deficiency in heart failure are not yet fully clarified. However, several contributing factors have been proposed, including impaired duodenal absorption due to intestinal edema, insufficient dietary iron intake, interactions with various medications, chronic gastrointestinal bleeding, and coexisting conditions such as chronic kidney disease [135,136]. Accurately assessing iron metabolism and diagnosing iron deficiency in heart failure remains challenging. Despite this, bone marrow biopsy is considered the gold standard for evaluating iron stores in the body and confirming a diagnosis of absolute iron deficiency. Nonetheless, it is an invasive procedure that is rarely used, and it is being replaced by the quantification of levels of several blood biomarkers, allowing us to determine iron status and make ID diagnoses [125,126,127,137]. They are briefly discussed below.

#### 5.1.1. ID Blood Biomarkers

Key biomarkers used to assess iron status include ferritin, **transferrin saturation (TSAT), soluble transferrin receptor (sTfR)**, and **hepcidin [138]. Ferritin** is an iron storage protein primarily produced by the liver and the reticuloendothelial system, and its serum levels generally reflect the amount of iron stored in body tissues. However, in the context of heart failure, ferritin levels may be elevated nonspecifically because of systemic inflammation, which can complicate the interpretation of iron status [138,139,140,141].

**Transferrin saturation (TSAT)**, the percentage of transferrin bound to iron, can be used as a parameter for quantifying the amount of iron available to cells. Malnutrition and catabolic metabolism can determine a reduction in serum transferrin than serum iron, resulting in a false increase in TSAT. Transferrin saturation (TSAT) and ferritin are commonly used markers to assess iron deficiency in clinical practice. According to the 2021 ESC guidelines for heart failure, iron deficiency should be treated in patients with heart failure when serum ferritin is below 100 µg/L, or when ferritin levels fall between 100 and 299 µg/L accompanied by a TSAT of less than 20% [142]. The usefulness of these two biomarkers in patients with heart failure has been investigated in a recent study, suggesting that the assessment of ferritin levels is not essential and that TSAT is a useful diagnostic tool in identifying patients with heart failure [138,143].

The **soluble transferrin receptor (sTfR)** is a membrane-bound receptor found in cells with a high iron demand. Its concentration in the bloodstream reflects both the number of these cells and the density of receptor expression, making it a reliable indicator of iron requirements and erythropoietic activity. Elevated sTfR levels suggest inadequate intracellular iron availability and have been linked to adverse clinical outcomes [138,143,144,145].

**Hepcidin** is a regulatory peptide hormone produced by the liver in response to pro-inflammatory signals, particularly via the interleukin-6 pathway. By binding to ferroportin 1 and promoting its lysosomal degradation, hepcidin inhibits iron release from enterocytes, hepatocytes, and macrophages into the bloodstream. Low circulating levels of hepcidin reflect depleted iron stores, regardless of the presence of anemia. Recent research has explored the combined use of low hepcidin and elevated **soluble transferrin receptor (sTfR)** levels as an alternative approach for diagnosing iron deficiency in heart failure. In a study involving 165 patients with acute heart failure, this combination effectively identified iron deficiency—capturing both iron store depletion and increased iron demand—and was also associated with worse prognosis in individuals with more severe deficiency [138,146,147].

In addition to the biomarkers abovementioned, **RDW**, a measure of the heterogeneity of the distribution of red blood cell size, has also been used to diagnose ID. High RDW values also occur in conditions of decreased availability of iron due to ID, anemia (low ferritin), or functional ID in the anemia of inflammation (AI) (normal/high ferritin). A study revealed that RDW is a parameter with a sensitivity of 94% and specificity of 59% in the diagnosis of ID [148,149].

#### 5.1.2. Clinical Viewpoint: Considerations and Limitations

Iron metabolism biomarkers in HF represent a perfect example of molecules with strong pathophysiological and clinical relevance for clinical application. Their value lies in the relationship between iron metabolism, pathophysiological bases, HF symptoms (e.g., exercise tolerance), and prognosis. Therefore, periodic screening for anemia and intellectual disability (ID) is strongly recommended for all HF patients, and any abnormalities should be identified as therapeutic targets in both acute and congestive HF [125,126,127].

When applying these biomarkers in clinical practice, it is essential to account for an important consideration. Historically, serum ferritin levels below 15–20 μg/L have been used to indicate iron deficiency at the level of the bone marrow. However, ferritin is also an acute-phase reactant and can be elevated in systemic inflammatory conditions, such as chronic kidney disease or heart failure, potentially obscuring true iron deficiency. As a result, roughly 25 years ago, diagnostic thresholds for ferritin were significantly increased—by approximately 5- to 20-fold—in patients with chronic kidney disease. Specifically, iron deficiency was defined as a ferritin level below 100 μg/L, or between 100 and 299 μg/L if transferrin saturation (TSAT) was below 20%. It is important to note, however, that this recommendation was not based on experimental evidence of total body or tissue iron depletion. Rather, it was introduced to promote iron supplementation and improve responsiveness to erythropoiesis-stimulating agents in individuals with renal anemia [138,150]. However, in the context of heart failure, this definition of iron deficiency is deemed inadequate, as it fails to effectively distinguish between absolute and functional iron deficiency. For instance, it may classify patients with normal serum ferritin levels (20–100 µg/L) and TSAT ≥ 20% as iron-deficient, even though they are not, often have a better prognosis, and typically do not benefit from iron supplementation. Moreover, in heart failure, serum ferritin concentrations can be influenced by certain therapies, such as neprilysin inhibitors and sodium–glucose co-transporter 2 (SGLT2) inhibitors, which are known to mobilize internal iron stores [138,150]. In contrast, the definition that appears more appropriate and confirmed by clinical trials, is hypoferremia, represented by a TSAT < 20%. Patients with such conditions respond to intravenous iron therapy, showing meliorated tolerance to the exercise, good functional capacity (when significantly impaired), and a pronounced decrease in the death or hospitalization risk (i.e., 20–30%). As a result, it has been suggested that the current ferritin-based definition of iron deficiency in heart failure should be replaced with a simpler, more accurate criterion focused solely on hypoferremia—defined as a transferrin saturation (TSAT) below 20% [125,126,127].

### 5.2. Altered Renal Function: Related Biomarkers

Renal function indicators, including **serum creatinine** and **glomerular filtration rate (GFR)**, are routinely employed in clinical practice to assess the response to heart failure therapy, particularly in relation to diuretic effectiveness [151], but also as prognostic biomarkers. In a study of 9289 patients with chronic HF, GFR was estimated based on creatinine levels, and was demonstrated to have independent and additive prognostic value compared to NT-proBNP and hs-TnT [152,153]. Several studies have identified cystatin C—a cysteine protease inhibitor that is widely expressed and exclusively cleared by glomerular filtration—as a potential prognostic marker in both acute and chronic HF with evidence suggesting it may outperform creatinine. Given the frequent coexistence of renal dysfunction in heart failure, additional biomarkers have been investigated. Among them, **neutrophil gelatinase-associated lipocalin (NGAL)**, a siderophore-binding protein released by neutrophils and epithelial cells in response to acute kidney injury and inflammation, has emerged as one of the most promising indicators [154]. In a cohort study involving 121 patients with acute heart failure, elevated NGAL levels exceeding 167.5 ng/mL (the 75th percentile) were associated with a 2.7-fold increased risk of mortality and a 2.9-fold higher risk of rehospitalization. Additionally, **kidney injury molecule-1 (KIM-1)** and **N-acetyl-β-D-glucosaminidase (NAG)**—both of which are expressed in proximal tubular epithelial cells—have been found to correlate with more severe NYHA functional class and reduced left ventricular ejection fraction in chronic heart failure. These findings suggest their potential utility as biomarkers for cardiorenal syndrome and indicators of prognosis in HF [154]. Notably, in an analysis involving 2130 participants from the GISSI-HF (Italian Group for the Study of Survival in Heart Failure) trial, NGAL, KIM-1, and NAG were each found to be independent predictors of the composite outcomes of mortality and heart failure-related hospitalization, regardless of baseline renal function [154].

#### Clinical Viewpoint: Considerations and Limitations

Such biomarkers are fundamental for HF prognosis. Therefore, the regular monitoring of renal function and treatment effectiveness is highly recommended for all patients with HF.

### 5.3. Altered Hepatic Function: Related Biomarkers

In patients with advanced HF, it is common to find an alteration of liver function. This depends primarily on the congestion of the hepatic venous system resulting from the right ventricular dysfunction. Consequently, some indices of liver dysfunction, such as increased **transaminases** and **bilirubin** and **hypoalbuminemia** (the latter also dependent on an enteric loss secondary to intestinal congestion), can stratify patients with acute or chronic HF at greater risk of adverse events [131].

### 5.4. Endocrine–Metabolic Changes

**Endocrine–metabolic changes** partly indicate the state of cachexia that occurs in patients with HF and contribute to its progression by increasing neurohormonal activation and inflammation. In individuals with chronic HF, thyroid dysfunction—often presenting as subclinical hypothyroidism or low T3 syndrome—is frequently observed and correlates with more severe clinical manifestations and poorer outcomes. Additionally, elevated serum and salivary cortisol concentrations are commonly reported and have been linked to increased mortality risk. Among adipokines, which regulate glucose and lipid metabolism, adiponectin is the most extensively studied. Notably, its levels rise in proportion to heart failure severity—despite being secreted inversely relative to body fat—and have been associated with unfavorable prognostic implications [153,155].

### 5.5. Bone Marrow Alterations: Decreased Levels of Circulating Endothelial Cells (CECs) and Endothelial Progenitor Cells (EPCs) in HF as Biomarkers of Negative Outcomes

Both patients with acute HF and, particularly, those with chronic HF, exhibit higher levels of inflammatory markers, as extensively described previously. Systemic inflammation is known to be significantly associated with reduced cardiac and vascular regenerative capacity, as well as impaired bone marrow function. The latter leads to reduced release of highly regenerative stem cells (i.e., EPCs) [156,157,158]. **Endothelial progenitor cells (EPCs)**, derived from the bone marrow, play a critical role in vascular repair, neovascularization, and maintaining endothelial integrity. Emerging evidence indicates that systemic inflammatory conditions, such as cardiovascular diseases—including heart failure—are associated with significantly reduced circulating levels of both EPCs **and circulating endothelial cells (CECs)**. These CECs, representing mature endothelial cells shed from the vascular lining, serve as indicators of vascular injury. The consistent observation of diminished EPC and CEC counts in patients with cardiovascular disorders has established their potential as circulating biomarkers of endothelial damage and impaired vascular repair mechanisms. Notably, patients with heart failure, particularly those with reduced ejection fraction (HFrEF), exhibit significantly lower levels of EPCs and CECs compared to age-matched individuals without a diagnosis of cardiovascular disease. These data suggest that these cell populations may be potential biomarkers of adverse heart failure outcomes. Specifically, EPCs have been shown to be independent predictors of mortality in heart failure patients (hazard ratio, 5.0; *p* = 0.001) [159]. Furthermore, a significant association between EPCs and traditional HF biomarkers, such as NT-proBNP, has been demonstrated [160,161]. This suggests that EPCs have the potential to be used as heart failure biomarkers with prognostic and outcome implications. Further studies are needed to clarify the role of EPCs in HF. Strategies to enhance EPCs, such as exogenous stem cell therapies designed to enhance regenerative capacity in HF, also need further exploration.

### 5.6. Another Bone Marrow Alteration: Clonal Hematopoiesis of Indeterminate Potential (CHIP) as Additional HF Biomarker

Clonal hematopoiesis of indeterminate potential (CHIP) has attracted considerable attention in HF research because of its association with a worse prognosis and adverse outcomes. Thus, CHIP is also worth considering as an excellent biomarker of HF. Accordingly, CHIP has also been observed to be associated with the various types of alterations of HF and their etiologies. Precisely, some trials reported that patients with ischemic HFrEF had a CHIP prevalence of about 18.5% [162]. In addition, they also had significantly negative long-term outcomes compared to patients without CHIP [162,163,164]. CHIP has also been associated with HF in patients with non-ischemic HFrEF. In patients without a known history of acute coronary atherosclerotic syndrome, CHIP has demonstrated to predict the HFrEF onset [162,163,164]. CHIP also has outcome’s significance in patients with both ischemic and non-ischemic HFrEF. In such patients, CHIP is related to augmented risk of HF-death and HF-hospitalization, as well as the development of cardiogenic shock [164,165]. CHIP has also been demonstrated to be an independent risk factor for incident HFpEF [166]. Patients who have HFpEF and CHIP have been shown to have a defective cardiac function and prognosis. Thus, CHIP is significant as a risk factor not only for hematological magnificence, but also as a novel cardiovascular risk factor particularly in patients with HF. However, its clinical efficacy is restricted until now. Used in combination with other prognostic factors and above-described HF outcome biomarkers, rapid advances in the clinical management of HF might be achieved and gene sequencing technology may be of help.

## 6. Considerations: Towards the Development of Multi-Biomarkers Panels Through Artificial Intelligence and Multi-Omics?

The emerging biomarkers described above, of which there are many, encourage the development of multi-biomarker panels, reflecting the different pathophysiological pathways involved in the onset and progression of HF [167]. The reason for this interest lies in the high potential that such multi-biomarker panels offer in the clinical context of a disease, such as HF. In theory, they would not only allow us to personalize and improve the clinical management of HF but also enable us to optimize the therapeutic strategy. Indeed, the possible increase or reduction in blood levels of a specific biomarker, detected using such panels, could offer us important information that we can translate into the context of a treatment by suggesting introducing, reducing, or increasing a treatment aimed at counteracting the pathophysiological pathway related to such increase or reduction. Consequently, this could allow the better personalization of therapy, although this approach has not yet been tested in randomized controlled clinical trials [108,168,169,170].

In addition to this consideration, the positivity of such panels, correlated with the increase in levels of different biomarkers, could be used to develop prognostic scores for HF, as well as for screening to estimate the risk of developing HF in the general population [170]. Experimental evidence from multiple studies supports the use of multi-biomarker approaches for risk stratification in both acute and chronic heart failure, as well as for assessing the likelihood of HF development in the general population. For instance, one study proposed a prognostic model that combined hs-TnT and NT-proBNP, using threshold values adjusted according to eGFR categories, to improve risk assessment in patients with chronic heart HF [86]. Furthermore, in such studies, the choice of biomarkers to include in the models was generally discretionary. Recent sub-analysis of the TOPCAT (Treatment of Preserved Cardiac Function Heart Failure with an Aldosterone Antagonist) trial employed a machine learning methodology to develop a multi-biomarker panel—selected from 49 analytes—to predict clinical outcomes, including mortality, hospitalization, and readmission, in patients with heart failure with a HFpEF [171]. Machine learning (ML) offers a robust approach for the development of multi-biomarker panels [172]. Machine learning techniques are increasingly applied in heart failure management to support diagnosis, enhance risk assessment, optimize therapeutic strategies, and aid in drug development—enabling a more personalized and data-informed approach to patient care [173]. Machine learning algorithms are also utilized to enhance heart failure risk prediction by analyzing data from electronic health records (EHR), multi-omics platforms, and information collected through wearable devices [168]. Furthermore, it also applies the digital twin ‘model [174]. Digital twin models enable personalized simulations, allowing for the assessment of disease progression and the prediction of individual responses to therapy [174]. Thus, artificial intelligence (AI) is also revolutionizing HF management, as well as cardiac imaging and phenotyping algorithms [175]. For instance, AI-assisted imaging can evaluate left ventricular function, myocardial fibrosis, and diastolic dysfunction with higher precision than traditional manual methods. This approach facilitates the development of HFpEF phenotyping models using machine learning, which integrate clinical information, biomarkers, and imaging data to categorize patients into distinct pathophysiological subtypes, enhancing the identification of therapeutic targets [176]. However, the validation of these models across diverse imaging datasets (such as echocardiograms) and healthcare systems remain in progress. Advances in precision medicine—particularly through the integration of genomic, proteomic, and metabolic data—suggest that artificial intelligence could also aid in identifying heart failure phenotypes that are more likely to benefit from targeted therapies [176]. Furthermore, AI is playing an increasingly pivotal role in accelerating drug development by analyzing complex biological datasets, predicting drug–target interactions, and repurposing existing therapies. Its continued advancement—especially when integrated with digital health tools and adaptive clinical trial designs—holds promise for optimizing heart failure management and treatment. Nonetheless, challenges such as data bias, a lack of interpretability, and ethical implications must be carefully addressed to ensure equitable and clinically meaningful implementation [176].

### From the Application of Multi-Omics to the Identification of Further Emerging Biomarkers: Genetic, Genomic and Transcriptome Biomarkers

Probably, the application of omics technologies could also facilitate the development of multi-biomarker panels [96]. Omics has expanded over the past 20 years, allowing for more specific knowledge of disease pathophysiology at the molecular level, thus leading to identifying biomarkers and targets for targeted treatments [96]. Accordingly, omics technologies enable the comprehensive study of molecules and biological processes involved in disease, encompassing various fields such as genomics, transcriptomics, epigenomics, proteomics, metabolomics, and beyond [177]. Thanks to multi-omics approaches, vast amounts of data can now be generated and integrated, offering powerful tools for unraveling complex biological processes and the mechanisms underlying disease development. In the context of HF, multi-omics has recently contributed to the identification of key genetic and epigenetic determinants that influence disease onset and progression. For instance, specific single-nucleotide polymorphisms (SNPs)—genetic variants that can modulate an individual’s susceptibility, disease trajectory, and treatment response—have been linked to HF. In patients with HFpEF, notable genetic variants have been found in genes related to inflammatory and fibrotic pathways, such as the interleukin-6 receptor (IL6R) gene. These findings help shed light on the systemic inflammation and fibrosis that play central roles in HF pathophysiology [178]. Likewise, in HFrEF, certain single-nucleotide polymorphisms (SNPs) within the beta-adrenergic receptor gene (ADRB1), especially the Arg389Gly variant, have been associated with differential responses to beta-blocker therapy. The Arg389 allele is linked to heightened beta-adrenergic signaling, leading to stronger myocardial contractility and a greater therapeutic sensitivity to beta-blockers [179]. Moreover, variations in the angiotensin-converting enzyme (ACE) gene, particularly the insertion/deletion (I/D) polymorphism, have been found to significantly influence the renin–angiotensin–aldosterone system (RAAS). Specifically, individuals carrying the D allele tend to exhibit elevated circulating ACE levels, resulting in increased angiotensin II production. This cascade promotes myocardial remodeling and contributes to the progression of heart failure. As a result, patients with the DD genotype may experience greater benefit from ACE inhibitor therapy, underscoring the potential of tailoring RAAS-targeted treatments based on genetic profiles in HFrEF [180].

Beyond SNPs, cell-free DNA (cfDNA)—which is released into circulation during apoptosis or cellular necrosis—has emerged as a promising biomarker in heart failure. Elevated cfDNA levels have been correlated with myocardial injury and disease severity in HFrEF, as well as with endothelial dysfunction and microvascular impairment in HFpEF. When integrated with transcriptomic data, cfDNA profiling has the potential to enhance risk stratification and improve prognostic assessment, particularly in complex or borderline clinical presentations. Additionally, genome-wide association studies (GWAS) have identified genetic loci such as TTN and BAG3, which are linked to myocardial stress and fibrosis, offering further opportunities for refined risk prediction and the development of more personalized therapeutic strategies in heart failure management [181].

In addition to SNPs, transcriptomic elements—especially non-coding RNAs (ncRNAs)—have gained considerable interest for their regulatory functions in the development and progression of HF. Among these, microRNAs (miRNAs), a class of small endogenous ncRNAs, have been increasingly recognized for their critical role in post-transcriptional gene regulation. Recent research has highlighted their involvement in various molecular pathways associated with HF pathophysiology, underscoring their potential as both biomarkers and therapeutic targets [182]. They influence key processes such as infarct size modulation, cardiomyocyte repair, collagen deposition, and macrophage polarization. As a result, they may serve as specific biomarkers for cardiac hypertrophy and fibrosis. In HFpEF, miR-223 and miR-29 are clearly associated with myocardial fibrosis and systemic inflammation [182,183]; miR-503-5p and miR-193a-5p are promising biomarkers for the differential diagnosis between HFpEF and HFrEF [184,185]; miR-208 and miR-499 are associated with cardiac remodeling, myocardial stress and apoptosis in HFrEF cases (making them excellent indicators of disease severity and progression) [186]. miRNAs have also been proposed as potential therapeutic agents, given their ability to regulate key molecular pathways involved in heart failure pathogenesis [184]. Specifically, antimir and miRNA mimics can be used in different ways to control the expression of specific and circulating miRNAs; for example, they can be administered for the pathophysiological myocardial remodeling related to the onset of heart failure. Preclinical and early clinical studies have shown that these molecules are generally well tolerated and demonstrate therapeutic potential in both animal models and healthy human subjects. Their involvement in key regulatory processes positions microRNAs as promising candidates for diagnostic, prognostic, and therapeutic applications in heart failure. As such, their integration into clinical practice may support the development of more personalized and targeted treatment strategies. Some examples of miRNA in HF are reported in Table 3. Further studies are, however, needed, since this field is still in an embryonic form.

## 7. From Identifying New Molecules as Emerging Biomarkers to Applying Them as Targets for Innovative Treatments in HF

Conventional HF treatment primarily involves beta-blockers, angiotensin receptor–neprilysin inhibitors (ARNIs), and mineralocorticoid receptor antagonists (MRAs). However, despite their widespread use, these therapies often yield suboptimal clinical outcomes [176]. Recently, however, in both HFrEF and HFpEF cases, the use of sodium–glucose cotransporter 2 (SGLT2) inhibitors, soluble guanylate cyclase (sGC) stimulators, and cardiac myosin activators has demonstrated promising efficacy [176]. Recently, however, in both HFrEF and HFpEF, the use of sodium–glucose cotransporter 2 (SGLT2) inhibitors, soluble guanylate cyclase (sGC) stimulators, and cardiac myosin activators has demonstrated promising efficacy, as well as emerging molecularly targeted therapies such as CRISPR-Cas9 gene editing, RNA-based therapies, and adeno-associated virus serotype 9 sarcoplasmic reticulum calcium ATPase (AAV9-SERCA2a) gene therapy [176]. Some of these treatments are described below. Examples of their efficacy, highlighting their mechanism-specific interventions with respect to metabolic, contractile, and vascular abnormalities, are also reported.

### 7.1. Molecular Targeted Therapies

CRISPR-Cas9 gene editing offers the potential to correct or silence genetic variants, including mutations associated with various forms of dilated cardiomyopathy [187]. For example, truncating variants in the titin gene (TTNtv), as well as pathogenic mutations in myosin-binding protein C3 (MYBPC3), lamin A/C (LMNA), and BCL2-associated athanogene 3 (BAG3), represent some of the most common genetic alterations associated with the onset of dilated cardiomyopathies [187] that cause HF as complication. These mutations typically result in sarcomere dysfunction, thereby contributing to the development of HF. Preclinical studies, primarily conducted using induced pluripotent stem cell (iPSC)-derived cardiomyocytes, have demonstrated promising results [176]. However, several challenges remain before CRISPR-based therapies can be translated into clinical practice. These include concerns over off-target effects, immune responses to viral vectors, and the complexity of achieving efficient in vivo delivery. As alternatives, base editing and prime editing techniques offer more precise single-nucleotide corrections with minimal DNA damage, potentially enhancing safety profiles. In addition to gene editing strategies, adeno-associated virus serotype 9 (AAV9)-mediated gene therapy targeting SERCA2a has been explored to restore calcium homeostasis—a critical dysfunction in HF characterized by the downregulation of SERCA2a expression. The CUPID-1 (Calcium Upregulation by Percutaneous Administration of Gene Therapy in Cardiac Disease) trial showed that AAV9-mediated SERCA2a overexpression improved ventricular function. However, the subsequent CUPID-2 trial failed to replicate these findings, largely due to insufficient vector transduction efficiency [176].

Another promising targeted therapeutic approach involves RNA-based interventions. As previously noted, the use of antisense oligonucleotides (ASOs) and small interfering RNAs (siRNAs) represents a viable strategie for modulating gene expression in the context of HF. These molecules function by selectively silencing or modifying the expression of specific genes implicated in HF pathophysiology, offering the potential for disease-specific and personalized treatment options. Among miRNAs associated with HF, miR-132 emerges as a key regulator of cardiac remodeling and has been considered as an appropriate biomarker and treatment target of HF [176]. A recent trail, the Cardior-132-L (CDR132L), is currently in Phase 1, and is evaluating the treatments with specific ASOs and siRNAs to reduce the levels of miR-132 and simultaneously improve myocardial fibrosis and heart function. Another promising RNA therapy is inclisiran, known for reducing lipid levels. It might be applied to patients with HFpEF and systemic inflammation and dyslipidemia, limiting the atherosclerosis effects [176].

Another targeted therapeutic strategy in heart failure (HF) focuses on modulating inflammation to improve clinical outcomes. Anti-inflammatory interventions in HFpEF have shown encouraging results in recent clinical trials. For instance, treatment with biologics such as ziltivekimab has demonstrated a significant reduction in hs-CRP, along with improvements in vascular function and exercise tolerance [176]. Given the multifactorial nature of heart failure (HF) pathogenesis, a combinatorial therapeutic approach that integrates anti-inflammatory biologics with metabolic or vascular-targeted treatments may offer enhanced clinical benefits. Nevertheless, further research is necessary to validate this strategy. While these emerging therapies show significant promise, most remain in the experimental stages and are not yet part of standard clinical practice. In this context, the integration of artificial intelligence (AI) and multi-omics technologies holds potential to address existing limitations, particularly in patient stratification. Tools such as polygenic risk scores and transcriptomic profiling may improve the identification of appropriate candidates for personalized therapies, thereby optimizing treatment efficacy.

#### Challenges and Future Directions

Despite substantial advancements in pharmacological and molecularly targeted therapies for heart failure (HF), several critical challenges hinder their integration into routine clinical practice. Key issues include ensuring safety, scalability, cost-effectiveness, and real-world applicability. Long-term safety and efficacy remain major concerns: for instance, gene editing tools such as CRISPR-Cas9 may lead to off-target effects, immune responses, or unintended genomic alterations. Similarly, RNA-based therapies and adeno-associated virus (AAV)-mediated gene delivery require extended follow-up to assess durability and safety. The development of these therapies also demands the use of adaptive study designs and comprehensive long-term data. To overcome these barriers, innovative tools such as AI-driven platforms could play a crucial role by facilitating the translation of experimental strategies into personalized, effective, and widely accessible HF treatments.

### 7.2. Regenerative Therapy: EPCS as Promising Candidates of Progenitor Stem Cells Therapy

EPCs have been used as biomarkers of HF progression, risk and outcomes, as mentioned above. However, it is emerging that transplanted EPCs can mediate paracrine-signaling-induced effects on vascular remodeling, angiogenesis, and tissue repair in the treatment of CVDs, such as HF. However, further studies are needed to better understand the mechanisms of action of specific paracrine components (cytokines, proteins, and miRNAs), the specific cells targeted by EPCs, and the mechanisms of communication and signal transduction between EPCs and other cell types [156,157,158]. Alternative regenerative approaches are emerging as promising treatment strategies for heart failure (HF). These include engineered extracellular vesicles (EVs), such as exosomes derived from mesenchymal stem cells or cardiac progenitor cells, which act as cell-free therapeutic agents. These exosomes deliver microRNAs (miRNAs), growth factors, and anti-apoptotic molecules directly to the injured myocardium, promoting tissue repair and cardioprotection. Additionally, cardiomyocytes derived from induced pluripotent stem cells (iPSCs) and the development of 3D-bioprinted cardiac patches offer innovative avenues for myocardial regeneration and functional restoration in HF. Despite their well-documented efficacy, they show some limitations because they can cause immune rejection and graft integration. Additionally, their long-term viability is debated. Such limitations constitute key obstacles to successful clinical application. Further studies, requiring the support of multi-omics technologies, are needed [188,189,190].

## 8. Discussions and Conclusions: Considerations and Suggestions on the Evidence Described

Many biomarkers have been studied in HF, but their clinical application is still far from effective and largely insufficient, as most do not meet the criteria and therefore cannot yet be recommended. Further obstacles arise from the lack of standardized quantification criteria, the metric scales used, and the quality of the studies conducted, such as the number, the limited number of cases, the heart failure phenotypes considered, the exclusion of other comorbidities, and the clinical significance of individual or combined analyses. Conducting meta-analyses will likely provide a better understanding of the role of these analyses in heart failure, be it diagnostic, prognostic, outcome-related, or predictive, and form the basis for more complex definitions, such as guidelines that include panels of diagnostic, prognostic, and outcome biomarkers. Another aspect to consider, as previously highlighted, is the standardization of the tests used to quantify the analytes discussed to make the data produced more homogeneous. Therefore, it is essential to involve clinical and statistical aspects, such as sensitivity (Se) and specificity (Sp), used as measures of diagnostic accuracy, the analysis of the ROC (Receiver Operator Characteristic) curve, the preferred method for determining diagnostic accuracy, and the area under this curve (AUC), which indicate the diagnostic power of the test. Another aspect that should not be overlooked, but must be emphasized, is the fact that these biomarkers may lack specificity due to the elevated levels found in multiple inflammatory conditions beyond heart failure, such as infections, autoimmune diseases, chronic inflammatory disorders, and even acute stress. One solution to this issue is population standardization, given that HF is a syndrome frequently associated with other pathological conditions. Furthermore, baseline levels of inflammation vary from person to person. Genetic factors, lifestyle, and environmental exposures also play a role. To improve the specificity of this analysis, we suggest using a multimodal approach that considers the levels of these molecules along with other clinical, imaging, and physical examination data that demonstrate high clinical power, such as the patient’s medical history. Combining these data with the help of machine learning algorithms and considering the multimolecular characteristics of each individual patient allows for a more complete picture, leading to personalized treatments. Regarding innovative therapies, CRISPR-associated Cas9 gene editing and RNA-based therapies appear promising and are designed to address the genetic and inflammatory factors of HF. Regenerative medicine strategies present some challenges, but further research could be beneficial. Finally, AI, particularly through machine learning algorithms and advanced echocardiographic phenotyping, is revolutionizing the management of HF. By enhancing predictive modeling, risk stratification, and precision medicine, AI enables the integration of complex multi-omics datasets, thereby facilitating more personalized and effective therapeutic strategies.

Despite these promising progresses, diverse questions remain open. However, the future of HF management will depend on overcoming these current limitations through continued innovation, rigorous validation, and the integration of advanced technologies. Addressing challenges related to safety, scalability, cost-effectiveness, and clinical translation will be essential to fully realize the potential of emerging therapies and data-driven approaches. The path to achieving this goal is arduous, but achievable, and will require further efforts by researchers and clinicians with diverse multidisciplinary expertise.

## Figures and Tables

**Table 1:** The main advantages and limitations of clinical use of NT-proBNP.

Advantages	Limitations
Early diagnosis marker in patients with diabetes in the absence of a clear clinical expression of heart failure.	The increase in BNPs and NT-proBNPs may also depend on other comorbidities such as chronic renal failure or atrial fibrillation.
In the absence of a defined cardiovascular pathology, the dosage of NT-proBNP values could predict the onset of heart failure, coronary artery disease and stroke.	The value of NT-proBNPs should also be correlated with age, sex, and BMI.
NT-proBNP values are significantly associated with increased odds of advanced HF.	There is a significant “gray area” in which the diagnosis is rather indeterminate.
Correlation between NT-proBNP values and the risk of adverse events in patients with heart failure with preserved ejection fraction.	

**Table 2:** Association between HF phenotype and typical and atypical diagnostic biomarkers.

HF Phenotype	Typical Diagnostic Biomarker	Atypical Diagnostic Biomarker
**HFpEF**	NT-proBNP, MR-pro-ANP	MR-proADM, Gal-3, sST2, GDF-15, MMPs/TIMPs, FGF21, CRP
**HFmrEF**	NT-proBNP, MR-pro-ANP	chromogranin A, copeptin, sST2, CA125, CAR
**HFrEF**	NT-proBNP, MR-pro-ANP, cTn	chromogranin A, copeptin, sST2, CA125, CAR

**Table 3:** Some examples of miRNA in HF.

miRNA	Role
**mir-22**	Regulates calcium reuptake by sarcoplasmic reticulum Associated with hypertrophy and myocardial fibrosis
**miR-133/miR-223-3p**	Their silencing reduces GLUT4 expression and thus increases myocardial glucose uptake in HF patients
**miR-21**	Involved in HF-related fibrosis through the stimulation of the ERK-MAP pathway
**miR-1**	Involved in regulating myocardial hypertrophy
**miR-212/132**	Associated with cardiac hypertrophy and HF
